# Primary Lymphomas of the Female Genital Tract: Recognizing the Rare Mimicker of Gynecologic Malignancy

**DOI:** 10.3390/medsci14030409

**Published:** 2026-07-21

**Authors:** Sofoklis Stavros, Maria-Anastasia Daskalaki, Stefanos Dafopoulos, Efthalia Moustakli, Ismini Anagnostaki, Anastasios Potiris, Theodoros Karampitsakos, Konstantinos Dafopoulos, Georgios Daskalakis, Peter Drakakis

**Affiliations:** 1 Third Department of Obstetrics and Gynecology, University General Hospital “ATTIKON”, Medical School, National and Kapodistrian University of Athens, 12462 Athens, Greece; apotiris@med.uoa.gr (A.P.); theokarampitsakos@hotmail.com (T.K.); pdrakakis@med.uoa.gr (P.D.); 2 First Department of Obstetrics and Gynecology, Alexandra Hospital, Medical School, National and Kapodistrian University of Athens, 11528 Athens, Greece; anastasia.daskalaki00@gmail.com (M.-A.D.); gdaskalakis@yahoo.com (G.D.); 3 Department of Obstetrics and Gynaecology, Medical School, University of Patras, 26504 Patras, Greece; stefanosntf2001@gmail.com; 4 Department of Nursing, School of Health Sciences, University of Ioannina, 45500 Ioannina, Greece; ef.moustakli@uoi.gr; 5 Medical School, National and Kapodistrian University of Athens, 11528 Athens, Greece; isanagnostaki3@gmail.com; 6 Department of Obstetrics and Gynecology, Faculty of Medicine, School of Health Sciences, University of Thessaly, 41110 Larissa, Greece; kdafop@uth.gr

**Keywords:** diffuse large B-cell lymphoma, extranodal lymphoma, diagnostic imaging, image-guided biopsy, molecular profiling, immunochemotherapy

## Abstract

Primary lymphomas of the female genital tract (PLFGT) are extremely rare neoplasms, representing a minor fraction of both extranodal lymphomas and gynecologic malignancies. Due to their nonspecific clinical presentation and overlapping imaging features with more common gynecologic tumors, diagnosis is often delayed or missed. This narrative review aims to synthesize current evidence on the clinical characteristics, histologic subtypes, diagnostic approaches, treatment strategies, and prognostic factors of PLFGT, emphasizing recent developments that may influence clinical practice. A comprehensive literature review was conducted, incorporating data from institutional case series, population-based studies, and recent genomic investigations focusing on PLFGT across various anatomical sites: ovary, uterus, cervix, and vagina. PLFGT typically affects women aged 44–68 years, with the ovary being the most frequently involved organ. The most common subtype is diffuse large B-cell lymphoma (DLBCL), followed by Burkitt lymphoma and marginal zone lymphoma. Patients usually present with pelvic pain, mass, or abnormal bleeding, while B symptoms are infrequent. Image-guided core needle biopsy has emerged as a valuable diagnostic approach that may reduce unnecessary surgery. Characteristic sonographic findings, such as hypoechoic, well-defined lesions and homogeneous uterine echo reduction, should raise clinical suspicion, though primary and metastatic disease cannot be distinguished solely by imaging. Rituximab-containing regimens (R-CHOP) are the mainstay of treatment and have improved outcomes. Despite treatment, central nervous system (CNS) recurrence remains a concern, particularly in ovarian involvement. Additionally, mutations in MYD88 and CD79B, although not prognostic, offer potential for personalized therapy. Timely diagnosis and appropriate systemic therapy are critical for improving survival in PLFGT. Advances in imaging, biopsy techniques, and molecular profiling are reshaping the diagnostic and therapeutic landscape of this rare but clinically significant disease.

## 1. Introduction

Lymphomas comprise a heterogeneous group of hematological malignancies arising from the clonal proliferation of B lymphocytes, T lymphocytes, or natural killer (NK) cells at different stages of maturation [1,2]. Although lymphomas most commonly arise within lymph nodes, approximately one-third originate from extranodal sites, whereas involvement of the female genital tract is exceedingly uncommon, accounting for only 0.2–1.1% of extranodal non-Hodgkin lymphomas (NHLs) [3,4,5].

Primary lymphomas of the female genital tract (PLFGT) are rare neoplasms that may involve the ovary, uterus, cervix, vagina, and, less frequently, the fallopian tubes. The ovary is the most commonly affected organ, accounting for nearly half of reported cases, followed by the uterus and fallopian tubes [6]. The vast majority of PLFGTs are B-cell non-Hodgkin lymphomas, with diffuse large B-cell lymphoma (DLBCL) representing approximately 60–75% of cases, whereas follicular lymphoma (FL), extranodal marginal zone lymphoma of mucosa-associated lymphoid tissue (MALT lymphoma), Burkitt lymphoma, and peripheral T-cell lymphomas occur considerably less frequently [5,7,8].

An important clinical distinction should be made between primary and secondary genital tract involvement by lymphoma. Secondary involvement as part of disseminated systemic disease is more common, whereas true primary lymphomas are exceptionally rare and require careful exclusion of nodal or extranodal disease elsewhere. Diagnostic criteria initially proposed by Fox et al. remain an important historical framework for primary ovarian lymphoma, although contemporary diagnosis relies on comprehensive clinical, radiological, and pathological evaluation to establish the primary site of disease [9,10].

The diagnosis of PLFGT remains challenging because clinical manifestations are nonspecific and frequently mimic more common gynecologic malignancies. Consequently, many patients initially undergo surgery for presumed gynecologic cancer before the correct diagnosis is established, highlighting the importance of maintaining a high index of suspicion [11].

Accurate diagnosis requires histopathological examination with immunophenotypic characterization of tissue specimens. Imaging plays a complementary role in disease assessment, with fluorodeoxyglucose positron emission tomography/computed tomography (PET/CT) serving as the preferred modality for staging and evaluation of treatment response, while magnetic resonance imaging (MRI), computed tomography (CT), and ultrasonography provide valuable anatomical information [12,13,14,15,16,17,18]. Laboratory investigations, including complete blood count, serum lactate dehydrogenase (LDH), and, in selected high-risk patients, bone marrow examination and cerebrospinal fluid analysis, contribute to staging and risk assessment [15,16]. More recently, advances in molecular pathology and genomic profiling have identified recurrent alterations involving genes such as MYD88 and CD79B, improving our understanding of lymphoma biology and offering promising opportunities for personalized therapeutic approaches [19,20].

Management of PLFGT differs substantially from that of primary gynecologic malignancies. Systemic immunochemotherapy represents the cornerstone of treatment, whereas surgery is generally reserved for diagnostic purposes or the management of complications [21,22,23,24]. Prognosis depends largely on histological subtype, disease stage, and molecular characteristics [5].

Despite increasing recognition of PLFGT during the past decade, current evidence remains fragmented and is largely derived from retrospective institutional series, registry-based analyses, and isolated case reports because of the rarity of the disease. Consequently, many aspects of diagnosis, treatment, and prognostic stratification remain incompletely defined. This narrative review provides an updated overview of the epidemiology, clinicopathological features, diagnosis, treatment, and prognosis of PLFGT while highlighting current challenges and future directions for research.

## 2. Materials and Methods

This narrative review was conducted to provide a comprehensive overview of the current evidence regarding primary lymphomas of the female genital tract (PLFGT). Although the review was not designed as a systematic review, a structured literature search was performed to ensure broad coverage of the available evidence.

The definition of primary lymphoma of the PLFGT remains challenging because historical studies frequently applied heterogeneous diagnostic criteria, and some cases classified as primary would now be considered secondary involvement by systemic lymphoma. Throughout this review, the classification reported in the original studies was retained. Where available, contemporary diagnostic approaches, including comprehensive systemic staging with FDG-PET/CT and bone marrow evaluation when clinically indicated, were considered essential for distinguishing true primary disease from secondary genital tract involvement.

The literature search was conducted in the PubMed/MEDLINE database using the following search strategy: (“primary lymphoma”) AND (“female genital tract” OR “female genital system” OR “uterus” OR “ovary” OR “vagina” OR “cervix”). Only articles published in English during the previous 15 years were considered to reflect contemporary diagnostic and therapeutic approaches.

Original research articles reporting clinical, pathological, diagnostic, therapeutic, or prognostic data on PLFGT were considered eligible for inclusion. Review articles, case reports, conference abstracts, editorials, and publications without accessible full text were excluded. The inclusion and exclusion criteria are summarized in Table 1.

Given the rarity of PLFGT and the predominance of retrospective studies, institutional case series, and registry-based analyses, a narrative review was considered the most appropriate methodology to synthesize the available evidence. Consequently, a formal assessment of study quality or risk of bias was not performed. To illustrate the evolution of knowledge in this field, the included studies are presented in chronological order.

## 3. Results

### 3.1. Characteristics of the Included Studies

The available evidence on primary lymphomas of the female genital tract (PLFGT) consists predominantly of retrospective institutional series, multicenter cohort studies, registry-based analyses, and population-based investigations. Most studies originated from China, the United States, and Japan, although contributions from Europe, Africa, and India demonstrate the global occurrence of this rare malignancy. Sample sizes varied considerably, ranging from small case series of three patients to large registry analyses including more than 700 women. While institutional studies provided detailed clinicopathological and therapeutic data, population-based investigations offered valuable epidemiological and survival information. Owing to the rarity of PLFGT, no prospective randomized studies were identified, and the available evidence was derived predominantly from retrospective observational research. The principal characteristics of the included studies are summarized in Table 2.

### 3.2. Epidemiology and Anatomical Distribution

Primary lymphomas of the female genital tract are exceptionally rare malignancies that account for only a small proportion of extranodal non-Hodgkin lymphomas. Across the included studies, the ovary consistently emerged as the most frequently affected anatomical site, followed by the uterus and cervix, whereas primary vaginal lymphoma represented the least common localization [28,33,43,48]. Large population-based studies from the Surveillance, Epidemiology, and End Results (SEER) database reported ovarian involvement in approximately 37–39% of cases, followed by cervical (21–22%) and uterine disease (16–17%) [33,43]. Similarly, the recent multicenter Chinese study involving 724 women identified the ovary as the predominant site (30.7%), followed by the uterus (23.5%) [48].

The disease predominantly affects middle-aged and older women, with the median age at diagnosis ranging between 44 and 68 years in most institutional series [25,28,37]. Nevertheless, younger women may also develop PLFGT, particularly those diagnosed with Burkitt lymphoma, which has been reported more frequently in pediatric and adolescent populations, especially in endemic regions of West Africa [34,43]. Geographic differences in histological distribution have also been observed, with Burkitt lymphoma occurring more frequently in African populations, whereas diffuse large B-cell lymphoma predominates in Asia, Europe, and North America [30,33,34].

### 3.3. Histopathological Characteristics

Diffuse large B-cell lymphoma (DLBCL) was consistently identified as the predominant histological subtype across virtually all included studies, accounting for approximately 60–72% of primary lymphomas of the female genital tract (PLFGT) [28,33,37,43,48]. This predominance was observed irrespective of the anatomical site of origin, including the ovary, uterus, cervix, and vagina, confirming DLBCL as the principal pathological entity encountered in clinical practice [6,33,43]. The high prevalence of DLBCL has important therapeutic implications, as most patients are candidates for rituximab-based immunochemotherapy.

Follicular lymphoma (FL) represented the second most common histological subtype, accounting for approximately 11–13% of cases in large population-based studies [33,43,48]. Although FL may arise throughout the female genital tract, it appears to have a particular predilection for the lower genital tract, especially the cervix and vagina. The recent clinicopathological study by Saksena et al. demonstrated that follicular lymphoma of the lower female genital tract frequently exhibits an indolent clinical course with excellent long-term outcomes, reporting a 5-year overall survival of 100% and a relapse-free survival of 76% [44].

Burkitt lymphoma represented a smaller proportion of PLFGTs but demonstrated distinct epidemiological and clinical characteristics. It predominantly affected younger women and pediatric patients and was encountered more frequently in ovarian tumors and populations from endemic regions, particularly West Africa [33,34,43]. Despite its aggressive biological behavior, favorable outcomes may be achieved with prompt diagnosis and intensive chemotherapy [45].

Less frequently encountered histological subtypes included extranodal marginal zone lymphoma of mucosa-associated lymphoid tissue (MALT lymphoma), plasmablastic lymphoma, ALK-positive large B-cell lymphoma, lymphoplasmacytic lymphoma, peripheral T-cell lymphoma, and other rare B-cell neoplasms [30,32,46]. These uncommon entities contribute to the histopathological heterogeneity of PLFGT and may require individualized diagnostic evaluation and treatment strategies. In particular, plasmablastic lymphoma and ALK-positive large B-cell lymphoma have mainly been reported in immunocompromised patients, especially those with human immunodeficiency virus (HIV) infection, suggesting an association between immune dysfunction and the development of aggressive extranodal lymphomas [30].

Recent molecular studies have expanded the understanding of PLFGT biology. Recurrent mutations involving MYD88 and CD79B, both associated with activation of the NF-κB signaling pathway, have been identified in a substantial proportion of DLBCL cases arising in the female genital tract [35]. Although these alterations have not consistently demonstrated independent prognostic significance, they represent promising biomarkers for molecular classification and may provide opportunities for the development of targeted therapeutic approaches in selected patients [35,38].

Overall, the available evidence demonstrates that PLFGT comprises a heterogeneous group of lymphoid neoplasms with distinct pathological, molecular, and clinical characteristics. Nevertheless, the overwhelming predominance of DLBCL across anatomical sites underscores the importance of prompt histopathological diagnosis and appropriate immunophenotypic characterization to guide optimal therapeutic management.

### 3.4. Clinical Presentation

The clinical presentation of PLFGT is highly variable and often nonspecific, frequently resembling more common gynecologic malignancies. Consequently, diagnosis is frequently delayed, with many patients initially managed for presumed ovarian, uterine, or cervical cancer before histopathological confirmation of lymphoma [6,28,32,33].

Across the included studies, the most common presenting symptoms were abdominal or pelvic pain, abdominal distension, pelvic or adnexal masses, and abnormal vaginal or uterine bleeding [6,25,28,29,32,33,37,40,47]. Less frequent manifestations included vaginal discharge, dyspareunia, lower extremity edema, bowel or urinary symptoms due to mass effect, and constitutional (“B”) symptoms such as fever, night sweats, and weight loss [30,31,37,40]. Although B symptoms are considered characteristic of systemic lymphomas, they were relatively uncommon among patients with PLFGT and were generally associated with advanced-stage disease or aggressive histological subtypes [27,32,37].

Clinical manifestations may vary according to the primary anatomical site. Ovarian lymphomas usually present with abdominal pain, pelvic masses, abdominal bloating, or ascites and are frequently indistinguishable from primary epithelial ovarian cancer on clinical examination alone [25,27,28,29]. Uterine and cervical lymphomas more commonly manifest with abnormal uterine or postmenopausal bleeding, whereas vaginal lymphomas typically present with vaginal discomfort, bleeding, dyspareunia, or a palpable vaginal mass [30,37,39]. Because these symptoms overlap substantially with those of benign gynecologic disorders and primary gynecologic malignancies, clinical findings alone are insufficient to establish the diagnosis.

Several laboratory abnormalities have been reported in patients with PLFGT. Elevated serum lactate dehydrogenase (LDH) was one of the most consistent findings and was frequently associated with advanced disease burden and poorer prognosis [29,31,37,47]. Increased serum cancer antigen 125 (CA-125) levels were also commonly observed, particularly in patients with ovarian involvement, although this marker lacks specificity and may lead clinicians toward an incorrect diagnosis of epithelial ovarian carcinoma [26,29,32,40,47]. Elevated soluble interleukin-2 receptor (sIL-2R) levels have also been reported in selected studies and may reflect increased tumor activity [31,37].

Certain clinical features may provide clues that distinguish PLFGT from more common gynecologic cancers. The relative absence of constitutional symptoms, the presence of homogeneous bulky pelvic masses, elevated LDH disproportionate to CA-125 levels, and limited evidence of tissue necrosis or invasion on imaging should prompt consideration of lymphoma in the differential diagnosis [32,37,40]. Moreover, immunocompromised patients, particularly those with human immunodeficiency virus (HIV) infection, appear to be at increased risk of developing aggressive extranodal subtypes such as plasmablastic lymphoma, emphasizing the importance of careful clinical evaluation in this population [30].

Overall, the available evidence demonstrates that PLFGT lacks a characteristic clinical presentation. Maintaining a high index of suspicion in women presenting with atypical pelvic masses or unexplained gynecologic symptoms is therefore essential to facilitate early diagnosis, avoid unnecessary extensive surgery, and ensure timely initiation of appropriate systemic therapy.

### 3.5. Diagnostic Evaluation

The diagnosis of PLFGT remains challenging because of their rarity and the absence of pathognomonic clinical or radiological findings. In most patients, the initial clinical impression is that of a primary gynecologic malignancy, particularly ovarian, uterine, or cervical carcinoma, often resulting in unnecessary surgical intervention before the correct diagnosis is established [6,28,32,33]. Therefore, accurate diagnosis requires the integration of clinical assessment, imaging studies, histopathological examination, immunophenotyping, and appropriate systemic staging.

Imaging plays a fundamental role in the initial evaluation of suspected PLFGT. Ultrasonography is usually the first-line imaging modality and may reveal large, homogeneous, hypoechoic masses with well-defined margins and increased vascularity on Doppler examination [40]. Ovarian lymphomas frequently appear as bilateral solid masses, whereas cervical and uterine lymphomas typically demonstrate diffuse enlargement with relatively preserved anatomical architecture [17,18,40]. Although these findings may raise suspicion for lymphoma, they are not sufficiently specific to distinguish primary from secondary genital tract involvement or from other gynecologic malignancies [40].

Cross-sectional imaging further contributes to disease characterization and staging. Computed tomography (CT) typically demonstrates homogeneous soft-tissue masses with mild to moderate contrast enhancement, while magnetic resonance imaging (MRI) provides superior assessment of local tumor extent and adjacent organ involvement [17,18]. Fluorodeoxyglucose positron emission tomography/computed tomography (FDG-PET/CT) has become an essential component of the diagnostic workup because of its high sensitivity for detecting extranodal disease, evaluating treatment response, and identifying occult systemic involvement, thereby facilitating accurate staging and differentiation between primary and secondary lymphoma [12,13,14].

Despite advances in imaging, definitive diagnosis relies on histopathological examination. Tissue biopsy remains the gold standard, with immunohistochemical characterization required to establish the lymphoma subtype and guide subsequent treatment [19,20]. Accumulating evidence supports the use of minimally invasive image-guided core needle biopsy whenever technically feasible, as it provides sufficient tissue for histopathological and immunophenotypic characterization while reducing unnecessary surgical intervention [31,32]. In contrast, superficial biopsies, particularly in vaginal lymphoma, may yield false-negative results because the neoplastic infiltrate often involves deeper stromal tissues while sparing the overlying epithelium. Likewise, cervical cytology frequently remains negative despite extensive stromal involvement, emphasizing the importance of deep tissue biopsy and histopathological evaluation when clinical suspicion persists [40,49,50].

Following histological confirmation, comprehensive systemic evaluation is essential to distinguish primary from secondary genital tract lymphoma. This evaluation generally includes whole-body FDG-PET/CT, bone marrow examination, complete blood count, serum lactate dehydrogenase (LDH), and other biochemical investigations [12,13,14,15,16]. Cerebrospinal fluid analysis should also be considered in selected patients at increased risk of central nervous system (CNS) involvement, particularly those with aggressive histological subtypes or high-risk clinical features [16,38].

**Figure 1:**
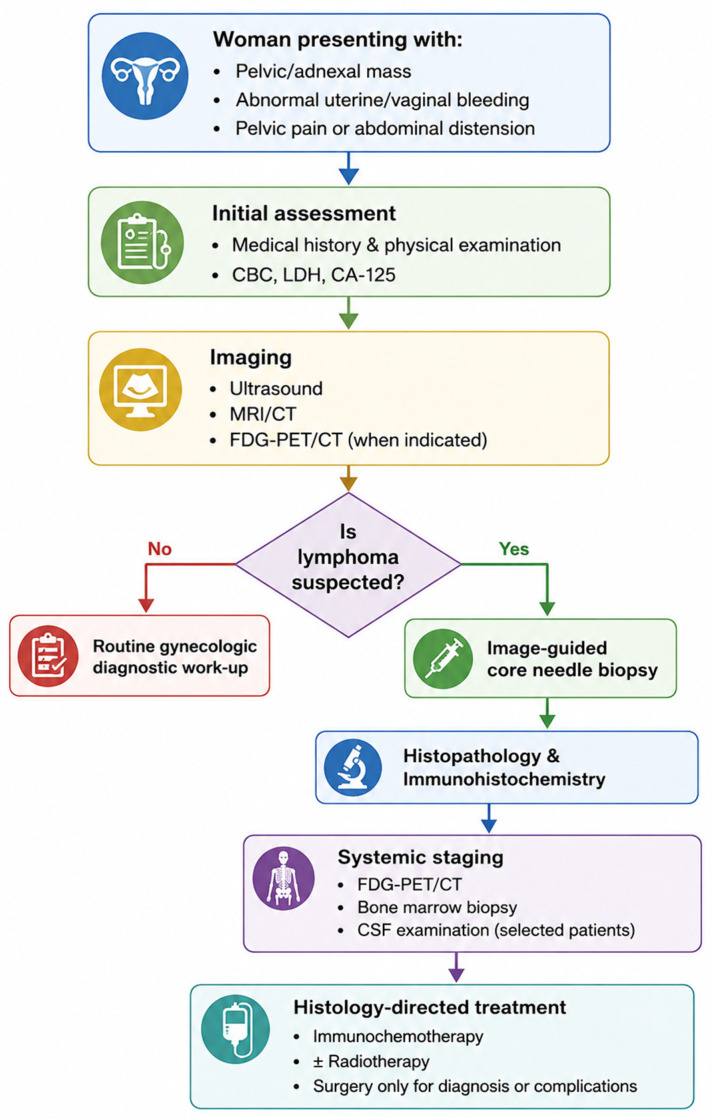
Suggested diagnostic algorithm for suspected primary lymphoma of the female genital tract (PLFGT).

Recent advances in molecular pathology have further refined the diagnostic evaluation of PLFGT. Recurrent alterations involving MYD88 and CD79B, particularly in diffuse large B-cell lymphoma, have improved the understanding of disease biology and may facilitate molecular classification and future targeted therapeutic approaches [35,38]. Although these biomarkers have not yet demonstrated consistent prognostic significance, they represent promising tools for precision oncology and may become increasingly relevant as molecularly targeted therapies continue to evolve. Based on the available evidence, a proposed diagnostic algorithm for the evaluation of women with suspected primary lymphoma of the female genital tract is presented in Figure 1.

Overall, early recognition of atypical clinical and imaging findings, followed by prompt tissue biopsy and multidisciplinary evaluation, is essential for establishing an accurate diagnosis. Such an approach minimizes unnecessary radical surgery, facilitates timely initiation of systemic therapy, and may ultimately improve clinical outcomes in this rare but clinically significant group of malignancies.

### 3.6. Treatment Strategies

The management of primary lymphomas of the female genital tract (PLFGT) requires a multidisciplinary approach involving gynecologic oncologists, hematologists, pathologists, and radiation oncologists. Unlike epithelial gynecologic malignancies, treatment is primarily determined by the histological subtype, disease stage, and patient-related factors rather than the anatomical site of origin. Consequently, systemic therapy represents the cornerstone of PLFGT management, whereas surgery is generally not considered a curative modality and is primarily performed for diagnostic purposes or the management of complications [22,23].

Diffuse large B-cell lymphoma (DLBCL), the predominant histological subtype, is most commonly treated with rituximab-based immunochemotherapy. The combination of rituximab, cyclophosphamide, doxorubicin, vincristine, and prednisone (R-CHOP) remains the standard first-line regimen and has been associated with favorable response rates and improved long-term survival across numerous institutional and population-based studies [6,22,28,37,46,47]. Patients diagnosed before the introduction of rituximab generally experienced poorer outcomes, highlighting the substantial survival benefit associated with modern immunochemotherapy [42,46]. In selected high-risk patients, particularly those with ovarian involvement or an increased risk of CNS relapse, CNS-directed prophylaxis with high-dose methotrexate or intrathecal chemotherapy may also be considered, although the available evidence remains limited [16,38].

Treatment of less common histological subtypes is less well established because of their rarity. Follicular lymphoma generally demonstrates a more indolent clinical course and may be successfully managed with rituximab-containing chemotherapy, localized radiotherapy in selected early-stage cases, or combined treatment modalities depending on disease extent [33,44,46]. Burkitt lymphoma requires intensive multi-agent chemotherapy because of its highly aggressive biological behavior, although favorable outcomes can be achieved with prompt diagnosis and treatment [33,34,45]. Similarly, uncommon entities such as extranodal marginal zone lymphoma (MALT lymphoma), plasmablastic lymphoma, peripheral T-cell lymphoma, and ALK-positive large B-cell lymphoma require individualized therapeutic strategies based on current lymphoma-specific treatment guidelines and multidisciplinary decision-making [30,46].

The role of surgery in PLFGT has evolved considerably. Historically, many patients underwent hysterectomy and bilateral salpingo-oophorectomy because the disease was initially presumed to represent a primary gynecologic malignancy [25,28,29]. However, available retrospective evidence does not demonstrate a survival benefit from extensive surgical resection, and surgery should not be considered definitive treatment for lymphoma [6,23,32]. Instead, surgery should be limited to obtaining adequate tissue for diagnosis or managing complications such as hemorrhage, bowel obstruction, or ovarian torsion [23]. The increasing use of image-guided biopsy has further reduced the need for unnecessary radical surgical procedures [31,32].

Radiotherapy may serve as an adjunct to systemic therapy in carefully selected patients. It has primarily been used for localized disease, residual tumor following chemotherapy, or palliative symptom control, although its precise role remains uncertain because of the limited number of available studies [23,46,48]. Population-based analyses have suggested that combined chemotherapy and radiotherapy may improve cancer-specific survival in patients with early-stage DLBCL, whereas chemotherapy combined with surgery may provide better outcomes in selected patients with advanced disease [48].

Recent advances in lymphoma treatment have introduced several novel therapeutic approaches for relapsed or refractory disease. Chimeric antigen receptor T-cell (CAR-T) therapy and other targeted immunotherapies have demonstrated promising efficacy in aggressive B-cell lymphomas, including DLBCL. However, their application in PLFGT remains largely hypothetical and is extrapolated primarily from evidence in systemic aggressive B-cell lymphomas, as disease-specific data are extremely limited [24]. In addition, ongoing advances in molecular profiling may facilitate the future development of personalized therapeutic strategies targeting recurrent alterations such as MYD88 and CD79B, although their clinical applicability in PLFGT has yet to be established [35].

Overall, the available evidence supports systemic immunochemotherapy as the foundation of PLFGT management, with treatment individualized according to histological subtype, disease stage, molecular characteristics, and patient-specific factors. Because high-quality prospective studies are lacking, current recommendations continue to rely largely on retrospective institutional experience and established treatment principles for systemic lymphomas.

## 4. Discussion

Primary lymphomas of the female genital tract (PLFGT), although recognized as one of the rarest forms of extranodal non-Hodgkin lymphoma, remain a diagnostically challenging entity because distinguishing true primary disease from secondary genital tract involvement is often difficult [5,9,10,33,43,48]. Despite increasing recognition over the past two decades, the available evidence remains limited to retrospective institutional series, multicenter observational studies, and population-based registry analyses, reflecting both the rarity of these malignancies and the absence of standardized diagnostic criteria in older studies [28,33,41,43,46,48]. Nevertheless, several important clinical patterns emerge consistently across the literature and may assist clinicians in improving diagnostic accuracy and patient management.

One of the principal findings of this review is that PLFGT should always be considered in the differential diagnosis of atypical gynecologic masses. Although ovarian, uterine, cervical, and vaginal lymphomas differ in their anatomical presentation, they share a common pattern of nonspecific clinical manifestations that frequently mimic primary gynecologic malignancies [6,25,28,32,37,40]. Consequently, many patients historically underwent extensive surgical procedures before the correct diagnosis was established [25,28,29]. Increasing awareness of this entity, together with early image-guided biopsy and multidisciplinary evaluation, may reduce unnecessary surgery and allow prompt initiation of systemic therapy [31,32,40].

Another consistent observation across the included studies is the predominance of diffuse large B-cell lymphoma (DLBCL), which accounts for approximately two-thirds of reported cases [28,33,37,43,48]. This finding has important therapeutic implications because most patients are candidates for rituximab-based immunochemotherapy, which has improved outcomes compared with earlier treatment eras [22,42,46]. Nevertheless, less common histological subtypes, including follicular lymphoma, Burkitt lymphoma, extranodal marginal zone lymphoma, plasmablastic lymphoma, and peripheral T-cell lymphoma, highlight the biological heterogeneity of PLFGT and emphasize the need for individualized treatment according to histopathological subtype rather than anatomical site alone [30,33,34,44,46].

Diagnostic evaluation has also evolved considerably. Historically, diagnosis was frequently established after hysterectomy or oophorectomy performed for presumed gynecologic malignancy [25,28,29]. More recent evidence supports ultrasound- or CT-guided core needle biopsy as a reliable approach for obtaining diagnostic tissue while avoiding unnecessary radical surgery [31,32,39,40]. Furthermore, FDG-PET/CT is increasingly important for systemic staging, treatment response assessment, and differentiation between primary and secondary genital tract involvement [12,13,14]. This distinction is clinically relevant because secondary genital tract involvement is more common and has implications for staging, prognosis, and treatment planning [9,10].

Prognosis appears to depend predominantly on established lymphoma-related factors rather than the anatomical site alone. Across the reviewed studies, advanced disease stage, elevated LDH, older age, aggressive histological subtype, bulky disease, poor performance status, and absence of rituximab-based therapy were associated with inferior survival outcomes [33,37,43,46,48]. Conversely, patients with localized disease treated with modern immunochemotherapy frequently achieved durable remission, with several series reporting favorable long-term survival [28,42,44,46]. Although ovarian involvement has been associated with an increased risk of central nervous system relapse, current evidence remains insufficient to establish uniform recommendations regarding CNS prophylaxis [32,38].

Recent advances in molecular pathology have begun to improve understanding of PLFGT biology. Recurrent MYD88 and CD79B mutations, particularly in DLBCL, suggest overlap with other extranodal lymphomas and may provide opportunities for molecular classification and targeted therapy [35,38]. Although these alterations have not consistently demonstrated independent prognostic value, they represent promising biomarkers for future risk stratification and precision medicine approaches [35].

Overall, the evidence synthesized in this review indicates that successful management of PLFGT depends on early clinical suspicion, accurate histopathological diagnosis, appropriate systemic staging, and multidisciplinary management. However, the available evidence is limited by the predominance of retrospective studies and case reports, the absence of prospective clinical trials, and the lack of uniform diagnostic criteria across historical studies, all of which contribute to substantial heterogeneity in the published literature. While the rarity of the disease precludes large prospective trials, multicenter registries and molecular studies may progressively improve diagnostic accuracy, prognostic stratification, and individualized treatment strategies in this uncommon group of malignancies [46,48].

### 4.1. Diagnostic Challenges and Differential Diagnosis

One of the major challenges highlighted by the available literature is the considerable overlap between PLFGT and more common gynecologic malignancies. Patients frequently present with nonspecific symptoms, including pelvic pain, abnormal uterine bleeding, abdominal distension, or pelvic masses, while imaging findings often resemble epithelial ovarian, cervical, or uterine cancers [6,25,28,32,40]. Consequently, many women undergo unnecessary extensive surgery before lymphoma is considered in the differential diagnosis [25,28,29]. Increasing awareness among gynecologists, radiologists, and pathologists is therefore essential to facilitate earlier diagnosis and avoid inappropriate surgical management.

Another important diagnostic challenge is distinguishing true primary female genital tract lymphoma from secondary involvement by systemic lymphoma. Secondary genital tract involvement is considerably more common than primary disease, particularly in patients with disseminated lymphoma [9,10]. Moreover, the definition of PLFGT has evolved over time, and several historical studies classified patients with advanced-stage disease as primary genital lymphomas despite evidence of nodal or extranodal dissemination. This lack of uniform diagnostic criteria has contributed to substantial heterogeneity across the published literature. Contemporary diagnosis therefore relies on comprehensive systemic staging—including FDG-PET/CT, bone marrow evaluation when clinically indicated, and histopathological assessment—to accurately establish the primary site of disease and guide appropriate management [12,13,15].

### 4.2. Current Therapeutic Considerations

Although PLFGT may arise from different anatomical sites, the available evidence consistently demonstrates that treatment should be guided primarily by histological subtype rather than tumor location [22,46]. Diffuse large B-cell lymphoma remains the predominant subtype and is generally associated with favorable outcomes when treated with rituximab-based immunochemotherapy [6,22,28,37,46]. Conversely, less common subtypes—including Burkitt lymphoma, follicular lymphoma, MALT lymphoma, plasmablastic lymphoma, and peripheral T-cell lymphoma—require individualized treatment strategies according to established lymphoma-specific guidelines [30,33,34,44,46].

One of the most important changes in clinical practice has been the gradual shift away from extensive surgery. Historically, hysterectomy or bilateral salpingo-oophorectomy was frequently performed because PLFGT was mistaken for primary gynecologic carcinoma [25,28,29]. Current evidence indicates that surgery rarely improves oncological outcomes and should generally be limited to obtaining diagnostic tissue or managing complications such as hemorrhage or ovarian torsion [23,31,32]. The increasing use of image-guided biopsy has significantly reduced unnecessary surgical intervention while enabling earlier initiation of systemic therapy [31,32,40].

### 4.3. Prognostic Factors and Future Perspectives

Despite the rarity of PLFGT, several prognostic factors have been consistently identified across retrospective studies and population-based analyses. Advanced disease stage, elevated serum LDH, aggressive histological subtype, older age, bulky disease, poor performance status, and omission of rituximab-containing therapy have all been associated with inferior survival outcomes [33,37,43,46,48]. In contrast, patients with localized disease treated with contemporary immunochemotherapy frequently achieve durable remission and favorable long-term survival [28,42,44].

Recent advances in molecular pathology may further improve the management of PLFGT. Recurrent mutations involving MYD88 and CD79B, particularly in diffuse large B-cell lymphoma, support the concept that these tumors share molecular characteristics with other extranodal lymphomas and may benefit from future targeted therapeutic approaches [35,38]. Nevertheless, the current evidence remains insufficient to support routine molecularly guided treatment, highlighting the need for larger multicenter studies integrating genomic profiling with clinical outcomes.

Given the rarity of PLFGT, prospective randomized clinical trials are unlikely to be feasible. Future progress will therefore depend on international collaborative registries, multicenter observational studies, and standardized reporting of clinicopathological and molecular data. Such efforts may facilitate more accurate risk stratification, optimize treatment selection, and ultimately improve outcomes for women affected by these uncommon malignancies [46,48]. The clinical presentation, diagnostic approach, treatment, and prognosis of PLFGT vary according to the site of involvement, as summarized in Table 3.

## 5. Clinical Implications: When to Suspect a PLFGT

Although PLFGT are rare, early clinical suspicion is essential because timely diagnosis may prevent unnecessary radical surgery and facilitate prompt initiation of systemic therapy. Clinicians should consider PLFGT in the differential diagnosis of women presenting with unexplained pelvic or adnexal masses, particularly when imaging findings are atypical for epithelial gynecologic malignancies [54,55,56,57,58].

Several clinical and radiological features may increase the suspicion of PLFGT. Ovarian lymphoma should be considered in patients with bilateral, solid, homogeneous pelvic masses, especially when ultrasonography demonstrates hypoechoic lesions with relatively well-defined margins and mild to moderate vascularity [27,49,59]. Similarly, diffuse uterine enlargement with preservation of the endometrial architecture or homogeneous cervical enlargement without extensive tissue destruction should prompt consideration of lymphoma rather than primary gynecologic carcinoma [17,18,37,40].

Although serum CA-125 may be elevated, particularly in ovarian lymphoma, it lacks specificity and should be interpreted cautiously [26,29,32,40,47]. Conversely, elevated serum lactate dehydrogenase (LDH) and soluble interleukin-2 receptor (sIL-2R) levels may support the diagnosis of lymphoma, particularly in patients with advanced disease [29,31,37,47]. Constitutional (“B”) symptoms, including fever, night sweats, and unexplained weight loss, although relatively uncommon in PLFGT, should further increase clinical suspicion when present [27,30,37].

Because imaging findings frequently overlap with those of more common gynecologic malignancies, histopathological confirmation remains mandatory. Whenever technically feasible, image-guided core needle biopsy should be considered before definitive surgery, as it provides adequate tissue for immunohistochemical and molecular characterization while avoiding unnecessary hysterectomy or oophorectomy in many patients [31,32,39]. Deep tissue sampling is particularly important in vaginal lymphoma, where superficial biopsies may be falsely negative because the neoplastic infiltrate often spares the overlying epithelium [39,44].

Following histological confirmation, comprehensive systemic staging—including FDG-PET/CT, laboratory evaluation, and bone marrow examination when indicated—is essential to distinguish primary from secondary genital tract involvement and to guide appropriate treatment planning [12,13,14,15,16]. Ultimately, early multidisciplinary collaboration among gynecologists, hematologists, radiologists, and pathologists is fundamental to achieving accurate diagnosis, minimizing unnecessary surgical intervention, and optimizing patient outcomes.

## 6. Limitations of the Existing Literature

The available evidence regarding PLFGT remains limited because of the exceptional rarity of these malignancies. Most published studies consist of retrospective single-center series, registry-based analyses, and small observational cohorts, while prospective studies and randomized clinical trials are lacking. Consequently, the quality of the available evidence is generally low, and many conclusions are based on relatively small patient populations [33,41,44].

Another important limitation is the considerable heterogeneity among published studies. Differences in patient selection, histological subtypes, staging systems, treatment strategies, follow-up duration, and reported outcome measures make direct comparisons difficult and preclude robust evidence synthesis. Furthermore, historical studies did not consistently distinguish primary female genital tract lymphoma from secondary genital tract involvement. Because diagnostic definitions and staging procedures have evolved considerably over time, some patients classified as having primary disease in older reports would likely be considered to have secondary lymphoma according to contemporary diagnostic criteria. This limitation introduces potential classification bias and contributes to the heterogeneity of the available epidemiological, clinical, and survival data [33,41,46].

The rarity of PLFGT also makes publication bias more likely, as unusual clinical presentations and favorable treatment outcomes are more frequently reported than typical or unsuccessful cases. In addition, many institutional series span several decades, during which important advances in diagnostic imaging, immunohistochemistry, molecular characterization, and rituximab-based immunochemotherapy have substantially changed clinical practice. Therefore, outcomes reported in older studies may not accurately reflect current standards of care [4,33,41,48].

Despite these limitations, the available literature provides valuable insights into the epidemiology, clinicopathological characteristics, diagnosis, treatment, and prognosis of this uncommon disease. Continued international collaboration and standardized reporting of future cases will be essential to improve the quality of evidence and facilitate more reliable clinical recommendations [4,46].

## 7. Future Directions

Despite recent advances in the understanding of PLFGT, several important knowledge gaps remain. Given the rarity of these malignancies, future research should prioritize the establishment of international multicenter registries and collaborative networks to facilitate the prospective collection of standardized clinical, pathological, molecular, treatment, and outcome data. Such initiatives would improve epidemiological characterization, enable more robust prognostic modeling, and provide a stronger evidence base for clinical decision-making [41,44,46]. However, the successful implementation of these registries will require standardized data collection protocols, rigorous data quality assurance, harmonized diagnostic criteria, compliance with privacy and regulatory requirements, and sustainable long-term funding.

Future studies should also focus on the molecular landscape of PLFGT. Comprehensive genomic and transcriptomic profiling may identify novel diagnostic biomarkers, prognostic indicators, and actionable therapeutic targets. In particular, further investigation of recurrent molecular alterations, including MYD88 and CD79B mutations, may improve risk stratification and support the development of personalized treatment strategies for selected lymphoma subtypes [8,48,54].

The optimal management of PLFGT remains incompletely defined. Although randomized clinical trials are unlikely to be feasible because of the low incidence of these tumors, well-designed prospective observational studies and international registry-based analyses could provide valuable comparative data regarding the role of systemic immunochemotherapy, radiotherapy, conservative surgical approaches, fertility-preserving management in younger women, and central nervous system prophylaxis in high-risk patients [41,44,55].

Another important research priority is the development of standardized diagnostic algorithms that integrate clinical presentation, advanced imaging, histopathology, immunophenotyping, and molecular profiling. Such algorithms could facilitate earlier diagnosis, reduce unnecessary radical surgery, and promote more consistent management across institutions [12,41,56].

Finally, future investigations should incorporate patient-reported outcomes, health-related quality of life, fertility outcomes, and long-term survivorship into routine data collection. As survival continues to improve with modern immunochemotherapy, these patient-centered outcomes will become increasingly important for optimizing both clinical care and quality of life in women diagnosed with PLFGT. The development of consensus recommendations by international multidisciplinary expert groups may further contribute to harmonizing diagnostic and therapeutic approaches for these rare malignancies [57].

## 8. Limitations of the Present Review

Several limitations of the present review should be acknowledged. First, this study was intentionally designed as a narrative review to provide a clinically oriented synthesis of the available evidence regarding primary lymphomas of the female genital tract (PLFGT). Given the rarity of these malignancies and the predominance of retrospective observational studies, case series, and registry-based analyses, a quantitative synthesis or meta-analysis was not considered appropriate [6,46,58].

Second, although a structured literature search and predefined eligibility criteria were applied, only studies published in English and indexed in PubMed/MEDLINE were included. Consequently, relevant publications indexed exclusively in other databases or published in other languages may not have been captured.

Third, the included studies demonstrated substantial heterogeneity with respect to patient populations, histological subtypes, diagnostic criteria, staging systems, treatment strategies, follow-up duration, and reported outcome measures. This variability limited direct comparisons between studies and precluded quantitative data synthesis.

Finally, because most of the available evidence originates from retrospective studies conducted over several decades, the findings should be interpreted in the context of evolving diagnostic techniques, pathological classifications, imaging modalities, and therapeutic approaches, particularly following the introduction of rituximab-based immunochemotherapy. Nevertheless, by synthesizing the currently available evidence, this review provides a comprehensive and clinically relevant overview of the epidemiology, diagnosis, treatment, and prognosis of PLFGT while identifying important areas requiring further investigation.

## 9. Conclusions

PLFGT are rare extranodal malignancies that remain challenging to diagnose because of their nonspecific clinical presentation and their resemblance to more common gynecologic tumors. The available evidence highlights the importance of maintaining a high index of suspicion, obtaining timely histopathological confirmation, and performing comprehensive systemic staging to establish an accurate diagnosis and guide appropriate management. Current treatment is primarily based on systemic immunochemotherapy, while surgery should generally be limited to diagnostic purposes or selected complications. Although significant advances have been made in imaging, molecular pathology, and lymphoma-directed therapies, further international collaborative studies are needed to improve the understanding of PLFGT, optimize treatment strategies, and support the development of evidence-based clinical recommendations.

## Figures and Tables

**Table 1:** Inclusion and exclusion criteria.

Inclusion Criteria	Exclusion Criteria
Original primary research articles	Review articles
Full-text articles available	Case reports
Published in English	Non-English publications
Published within the previous 15 years	Articles without accessible full text

**Table 2:** Summary of published clinical studies on primary lymphomas of the female genital tract. The studies are presented in the order in which they were identified during the literature review.

Study	Country	Year	No. of Cases	Clinical Presentation	Stage at Diagnosis	Histology	Therapy	Survival
Zhao et al. [25]	China	1997–2006	14	Abdominal/pelvic pain, mass, ascites	IE (4), IIE (3), IVE (7)	DLBCL (13), lymphoblastic (1)	Surgery + chemo (incl. R-CHOP)	Median OS 23 mo
Hu et al. [26]	China	2012	3 cases	Abdominal pain; one HBsAg-positive; 1 developed CNS involvement	III, IV	DLBCL	Ovary biopsy; CHOP + Rituximab; intrathecal prophylaxis	2 CR, 1 with CNS relapse and death
Senol et al. [27]	Turkey	2014	5	Ovarian mass, pain, distention; 3 primarily ovarian	3 Stage I (primary), 2 systemic	DLBCL	Ovary biopsy/surgery; chemotherapy (+/−)	All diagnosed; 3 died, 2 showed CR in 6 and 12 months
Ahmad et al. [28]	USA	1980–2013	36	11% asymptomatic, 24% vague abdominal complaints, 14% B–symptoms	I (19), II–IV (17)	DLBCL (18), Burkitt (5), follicular (4), others (8)	Surgery + chemo (many R-CHOP) (+/−) radiotherapy	OS median 70 months, 10 diseased, 5 recurrences
Sun et al. [29]	China	1999–2012	14	Abdominal pain, distension; unilateral (10) or bilateral (4) ovarian mass	I(3), II(6), IV(5)	DLBCL (12), Burkitt (2); GCB subtype DLBCL; MYC/BCL2 rearrangements	Surgery ± chemo ± radiotherapy	8 died, 4 alive with disease, 2 disease-free after median 3–56 mo
Pather et al. [30]	South Africa	2015	6	Vaginal bleeding, weight loss, pelvic mass	Not stated	Plasmablastic (3), DLBCL (2), ALK + large B-cell lymphoma	Chemo (e.g., CHOP/R-CHOP)	Not specified
Rahman et al. [31]	Japan	2016	3	Pelvic mass, vaginal bleeding; mimicking gynecologic tumors	Not stated	DLBCL (all)	Surgery/biopsy + CHOP ± Rituximab	2 CR, 1 PR
Vijayakumar et al. [32]	USA	2016	5	Pelvic mass, sometimes with elevated CA-125; mimic gynecologic tumor	Not stated	DLBCL (5)	Surgery in 3 (unnecessary), biopsy in 2; CHOP ± Rituximab; intrathecal in CNS+	4 CR, 1 died from disease progression
Nasioudis et al. [33]	USA	1988–2012	697	Ovary (37%), cervix (21%), uterus (16%); often pelvic mass, ±CA-125	I (42.6%), II (17.9%)	DLBCL (59.8%), FL (11.9%), Burkitt, MALT, T-cell, others	Surgery in 62.8%, Chemo ± radiation	5-y OS: 70.2%, 5-y CSS: 75.2%; localized, young, FL better
Akakpo et al. [34]	Ghana	2001–2010	8	Lower abdominal mass or pain; Burkitt subtype common	Not stated	Burkitt lymphoma	Not clearly stated	Not reported
Cao et al. [35]	China	2017	12	Ovary or genital tract mass; study on mutations (MYD88/CD79B)	Not specified	DLBCL	Chemo ± Rituximab possibly; mutation analysis only	No survival impact from mutations
Boussios et al. [36]	Greece	–	n/a	Extranodal DLBCL including genital sites; symptoms varied	Varies	DLBCL	Likely R-CHOP ± RT	Not specified
Ishii et al. [37]	Japan	2000–2016	14	Vaginal bleeding (7), abdominal pain (3)	79% stage III/IV	DLBCL (72%), others	R-CHOP (7), CHOP (3), others (4); ±surgery/RT	86% CR (12/14); 3-yr OS 57.9%; 3 relapses; 5 died
Shen et al. [38]	China	2018	141 (FGS = 12)	Genital-tract masses; elevated LDH	Not site-specific	DLBCL	Chemo ± Rituximab; CNS prophylaxis focus	Median OS 28 mo (range 1–116); IPI ≤ 2, CR, Rituximab predictive; CNS relapse risk
Petrillo et al. [39]	Italy	2019	–(vaginal cases)	Vaginal mass, pain, bleeding	Not specified	Primary vaginal NHL (mostly DLBCL)	Biopsy/surgery; R-CHOP ± RT	Not specified
He et al. [40]	China	2020	99 total; genital FGS subset unclear	Genital masses; ultrasound hypoechoic lesions	Not specified	Likely DLBCL (part of broader PE-DLBCL group)	Chemo ± Rituximab; US diagnosis focus	Not specified
Turashvili et al. [41]	USA	2020	6 cervical lymphomas	Vaginal bleeding, cervical mass	Secondary involvement	DLBCL	Biopsy or cone/hysterectomy; chemo ± Rituximab	Not specified
Ensor et al. [42]	USA	1975–2017	223 (uterine DLBCL)	–	Compared uterine vs. non-uterine DLBCL	DLBCL (uterine)	Chemo-immunotherapy (R-CHOP era)	2-yr OS ~92.3%; uterine site had better OS vs. non-uterine
Peng et al. [43]	China	1975–2011 (SEER); 2011–2017 nomogram	617 PLFGT cases	General PLFGT pool	Age, histology, stage, therapy as prognostic factors	Mixed: DLBCL, FL, Burkitt, MALT	SEER treatments varied; nomogram-based R-CHOP, surgery, RT	Age, stage, histology, therapy independently predicted OS and DSS; developed nomograms
Saksena et al. [44]	USA	2000–2022	15 (lower FGT FCL)	Mass, bleeding, asymptomatic	Not stated	FL (80%), others	Chemo ± RT ± surgery	5-yr OS 100%, RFS 76%
Rajendran et al. [45]	India	1999–2017	3	Pain, mass, B-symptoms	Not stated	DLBCL (ovarian)	Surgery + R-CHOP	All achieved complete remission
Shi et al. [6]	China	2014–2022	13	Postmenopausal bleeding, pelvic mass, bloating	Stage II (6), IV (7)	DLBCL (12), FL (1)	Surgery (10), Chemo (10), ±RT	Median survival 32 mo; 8 alive, 3 died, 1 recurrence
Pirosa et al. [46]	Multicenter (7 countries)	1982–2012	60	Mostly ovarian and uterine involvement	Not detailed specifically; includes advanced FIGO stages	DLBCL (most frequent), FL, marginal zone lymphoma, Burkitt lymphoma, T-cell peripheral lymphoma	58 received chemotherapy (mono or combined with surgery/RT)	5-year survival 75%; 86% CR with systemic therapy only; 23/29 CR with combined therapy
Ali et al. [47]	Egypt	2016–2023	24 (15 PLFGT)	Abdominal pain; elevated CA-125 and LDH	Ann Arbor I: 7 patients; IV: 5 patients	DLBCL predominant	Surgery (9), chemotherapy (14), radiotherapy (1)	Remission rate 60%
Liu et al. [48]	China	2000–2021	724	Not detailed	49% limited stage (Ann Arbor I-II); 21% advanced (III-IV)	DLBCL (65.2%), FL (11%)	Surgery, chemotherapy, radiotherapy; treatment varied by stage	Incidence declining; survival influenced by age, pathology, treatment; combined CT + RT improved CSS in early stage; CT+surgery better in advanced stage

**Table 3:** Comparative overview of primary lymphomas of the female genital tract by anatomical site.

Parameter	Ovary	Uterus	Cervix	Vagina	References
Relative frequency	Most common site of PLFGT	Second most common site	Less common than ovary and uterus	Rarest anatomical site	[6,33,39,43,48]
Typical age at presentation	Wide age range (young adults to elderly); most commonly 45–65 years	Predominantly middle-aged and older women	Most commonly fourth to sixth decades	Usually around the fifth decade	[25,28,29,30,33,37,39,41,44]
Most common clinical presentation	Pelvic/abdominal pain, adnexal or pelvic mass, abdominal distension, ascites; B symptoms uncommon	Abnormal uterine or postmenopausal bleeding, enlarged uterus, pelvic pain	Vaginal bleeding, cervical mass, vaginal discharge, pelvic pain	Vaginal mass, bleeding, dyspareunia, vaginal discomfort or discharge	[25,26,27,28,29,30,31,32,33,37,39,41,42,44,45,47]
Predominant histological subtype	DLBCL; Burkitt lymphoma more frequent than at other sites	DLBCL; occasional MALT lymphoma	DLBCL; follicular lymphoma and other B-cell lymphomas	DLBCL	[25,28,31,32,33,34,37,42,44,45]
Diagnostic challenges	Frequently mimics epithelial ovarian carcinoma	May resemble endometrial carcinoma or uterine sarcoma	Often clinically indistinguishable from cervical carcinoma	Frequently mistaken for primary vaginal carcinoma or benign vaginal lesions	[25,28,29,30,31,32,33,37,39,41,42,44]
Preferred diagnostic approach	Image-guided core needle biopsy whenever feasible; surgery mainly for diagnosis or complications	Endometrial sampling or image-guided biopsy with histopathological confirmation	Cervical punch or cone biopsy with immunohistochemistry	Deep vaginal biopsy is preferred, as superficial biopsies may be falsely negative	[31,32,37,39,40,41,42,44]
Typical imaging findings	Bilateral solid hypoechoic masses with relatively homogeneous appearance; MRI/CT useful for staging	Diffuse uterine enlargement with relatively preserved architecture; MRI useful for assessing local extension	Diffuse cervical enlargement with preserved cervical architecture	Homogeneous vaginal mass with intact overlying mucosa; MRI/CT assists local staging	[17,18,39,40,41,42]
Laboratory findings	LDH frequently elevated; CA-125 may also be increased	LDH occasionally elevated	LDH may be elevated	Limited laboratory data; LDH may be elevated	[26,29,30,31,32,37,39,40,41,42,47]
Treatment	Histology-directed systemic therapy (most commonly R-CHOP for DLBCL); surgery primarily diagnostic; CNS prophylaxis considered in selected high-risk patients	Systemic immunochemotherapy; radiotherapy in selected localized cases; surgery has a limited role	Systemic immunochemotherapy with or without radiotherapy; surgery rarely indicated	Systemic immunochemotherapy with selective use of radiotherapy; surgery limited to biopsy	[22,23,28,32,33,37,38,39,41,42,44]
Prognosis	Generally favorable in localized disease; poorer outcomes associated with advanced stage or CNS involvement	Variable; delayed diagnosis and disseminated disease may adversely affect survival	Favorable when diagnosed early and treated appropriately	Limited evidence available; localized disease generally demonstrates good response to treatment	[28,32,33,37,38,39,41,44,46]
Key clinical message	Consider lymphoma in bilateral solid ovarian masses with elevated LDH and relatively preserved ovarian architecture	Diffuse uterine enlargement without typical endometrial destruction should raise suspicion	Persistent cervical enlargement despite negative cervical cytology should prompt deep cervical biopsy	Persistent vaginal mass or unexplained vaginal bleeding should prompt histopathological evaluation through deep tissue biopsy	[6,51,52,53]

## Data Availability

No new data were created or analyzed in this study. Data sharing is not applicable to this article.
